# Value of the HFA-PEFF diagnostic algorithms for heart failure with preserved ejection fraction to the inflammatory myopathy population

**DOI:** 10.1186/s13075-023-03131-6

**Published:** 2023-08-04

**Authors:** Yunjing Shi, Hao Zhang, Zeping Qiu, Yanjia Chen, Xiuxiu Su, Huihui Chi, Tienan Feng, Yue Sun, Honglei Liu, Xiaobing Cheng, Junna Ye, Hui Shi, Qiongyi Hu, Zhuochao Zhou, Jianfen Meng, Jialin Teng, Chengde Yang, Yutong Su, Wei Jin

**Affiliations:** 1 Department of Cardiovascular Medicine, Heart Failure Center, Ruijin Hospital, Ruijin Hospital Lu Wan Branch, Shanghai Jiao Tong University School of Medicine, No. 197 Ruijin Second Road, 149 S. Chongqing Road, Shanghai, 200025 People’s Republic of China; 2 Department of Rheumatology and Immunology, Ruijin Hospital, Shanghai Jiao Tong University School of Medicine, No. 197 Ruijin Second Road, Shanghai, 200025 People’s Republic of China; 3 Clinical Research Institute, Shanghai Jiao Tong University School of Medicine, Shanghai, China

**Keywords:** Idiopathic inflammatory myopathy, Heart failure with preserved ejection fraction, HFA-PEFF score, AMA-M2, Anti-SRP antibody

## Abstract

**Objectives:**

The HFA-PEFF score has been validated to hold great diagnostic and prognostic utility for heart failure with preserved ejection fraction (HFpEF). Idiopathic inflammatory myopathy (IIM) is recognized as one of the potential etiologies underlying HFpEF. Here, we intended to investigate the real prevalence of HFpEF in IIM via the HFA-PEFF score and explore the prognostic value of this score.

**Methods:**

Two hundred twenty IIM patients were enrolled for assessment. The cohort was divided into low, intermediate and high tertiles of the HFA-PEFF score. Spearman’s correlation analysis was used to explore the association between the score and disease activity. Chi-square test was applied to investigate the distribution discrepancy of HFA-PEFF tertiles among patients with different myositis-specific antibodies (MSAs) or myositis-associated antibodies (MAAs). Univariate and multivariate ordinal regression analyses were performed to screen risk factors for high HFA-PEFF scores. Survival curves were obtained using the Kaplan–Meier method and log-rank tests.

**Results:**

In total, 79 (35.9%), 107 (48.6%) and 34 (15.5%) patients were rated low, intermediate and high probability of HFpEF, respectively. The HFA-PEFF score correlated well with disease activity. Patients with positive AMA-M2 scored higher in the HFA-PEFF score (*p* = 0.011). During follow-up, patients with positive AMA-M2 or anti-SRP antibody developed an inclination towards concentric hypertrophy on echocardiography. Additionally, palpitation symptom, AMA-M2 positivity and elevated serum levels of LDH, cTnI were independent risk factors for high HFA-PEFF scores. Finally, a high-tertile HFA-PEFF score was related to lower overall survival rate (*p* < 0.001). Patients with positive AMA-M2 had poorer outcomes (*p* = 0.002).

**Conclusion:**

HFpEF was prevailing in IIM patients according to the HFA-PEFF score. The HFA-PEFF score correlated well with disease activity and held significant prognostic value. Patients with AMA-M2 antibody were prone to have poor outcomes.

**Supplementary Information:**

The online version contains supplementary material available at 10.1186/s13075-023-03131-6.

## Key messages

1. The HFA-PEFF score is a useful tool to diagnose HFpEF in patients with IIM.

2. The HFA-PEFF score correlates well with the core set measures of myositis disease activity.

3. Patients with AMA-M2 were prone to have higher HFA-PEFF scores and lower overall survival rate.

4. The HFA-PEFF score holds significant prognostic value for all-cause mortality in patients with IIM.

**Supplementary Information:**

The online version contains supplementary material available at 10.1186/s13075-023-03131-6.

## Introduction

Idiopathic inflammatory myopathy (IIM), collectively known as myositis, is a rare group of autoimmune diseases encompassing heterogeneous clinical phenotypes, including dermatomyositis (DM), polymyositis (PM), immune-mediated necrotizing myopathy (IMNM) and inclusion body myositis (IBM) [1]. Autoantibodies are present in up to 90% of myositis patients, mainly referring to myositis-specific autoantibodies (MSA) or myositis associated autoantibodies (MAA) [2]. MSA has become a cornerstone of the diagnosis, classification and prognosis prediction in recent years for its specific relation to distinct clinical phenotypes [3–5].

IIM could be muscle-specific or multiple-organ involved (including the skin, joints, gastrointestinal system, lungs and hearts) [6]. Cardiac involvement is recognized as an unfavorable prognostic factor in IIM [7], and congestive heart failure constitutes a major cause of death in IIM [8, 9]. Noteworthy, in contrast to clinically manifest heart failure with reduced ejection fraction (HFrEF), heart failure with preserved ejection fraction (HFpEF) is liable to be neglected in clinical practice though it actually takes up over half of the whole heart failure population and is causal for poor outcomes [10]. HFpEF is characterized by elevated cardiac filling pressures, diastolic dysfunction and concentric cardiac hypertrophy with preserved cardiac systolic function [11, 12]. Cardiovascular related systemic microvascular endothelial inflammation and non-cardiovascular coexisting conditions are vital mechanisms contributing to HFpEF [13]. IIM is exactly a non-cardiovascular source of inflammation for HFpEF [14, 15]. In addition, diastolic dysfunction, a vital feature of HFpEF, was reported to be the most common cardiac manifestation of IIM [16, 17]. Nonetheless, scarce studies were published on HFpEF in IIM patients due to a lack of awareness.

Moreover, the diagnosis of HFpEF remained challenging due to its heterogeneity that a simple biomarker strategy such as NT-proBNP would not suffice. Recently, the Heart Failure Association (HFA) of the European Society of Cardiology (ESC) proposed a score-based algorithm, Pre-test assessment, Echocardiography and natriuretic peptide, Functional testing, Final etiology (PEFF) score to aid in the early recognition of HFpEF. The algorithm comprehensively integrates cardiac functional, morphological, and biomarker domains. A total HFA-PEFF score ≥ 5 points is considered to be diagnostic of HFpEF, while a score ≤ 1 point is considered to rule out HFpEF. An intermediate score (2—4 points) is supportive for HFpEF but needs further assessment [11].

The diagnostic utility of the score has been validated in multiple HFpEF cohorts such as the Maastricht cohort, the Northwestern Chicago cohort and a Japanese cohort [18, 19]. Additionally, the HFA-PEFF scoring system was applicable to specific populations such as the late elderly people, the middle-aged general population, subclinical HFpEF and cardiac amyloidosis [20–22]. In parallel, though initially developed as a diagnostic tool, the score was discovered to hold additional prognostic value. It turned out higher HFA-PEFF scores were related to heavier symptom burden, more adverse cardiovascular events and higher overall mortality rate [23, 24].

In view that IIM is recognized as a specific etiology underlying HFpEF-like syndromes, it is sensible to introduce the sensitive and concrete HFA-PEFF score to the IIM population. Herein, we applied it to our IIM cohort to illustrate the prevalence of HFpEF and further investigate the prognostic value of this scoring system in IIM.

## Methods

### Patients

**Fig. 1:**
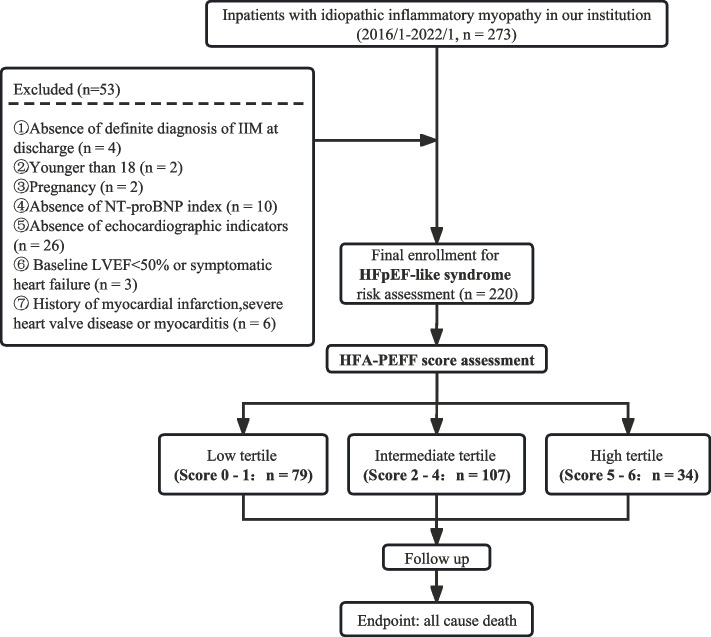
Flow chart of patient enrollment. *IIM* Idiopathic inflammatory myopathy, *NT-proBNP* N-terminal pro-B-type natriuretic peptide, *LVEF* Left ventricular ejection fraction, *HFpEF* Heart failure with preserved ejection fraction, *HFA-PEFF* Heart Failure Association Pre-test assessment, Echocardiography and natriuretic peptide, Functional testing, Final etiology

Two hundred seventy three inpatients diagnosed with IIM at the Department of Rheumatology and Immunology, Ruijin Hospital from January 2016 to January 2022 were reviewed and 220 patients were finally enrolled (Fig. 1). IIM was diagnosed according to the Bohan and Peter criteria or 2004 European Neuromuscular Centre (ENMC) criteria or the 2017 EULAR/ACR criteria [1, 25, 26]. The main exclusion criteria included: (1) absence of definite diagnosis of IIM at discharge, (2) younger than 18, (3) pregnancy, (4) absence of NT-proBNP index, (5) absence of echocardiographic indicators, (6) baseline LVEF < 50% or symptomatic heart failure, (7) history of myocardial infarction, severe heart valve disease or myocarditis. The study was performed in accordance with the Declaration of Helsinki and was approved by the independent Ethical Committees of Ruijin Hospital, Shanghai Jiao Tong University School of Medicine. Written informed consent was obtained from all participants.

### Clinical, laboratory, and imaging data collection

Demographic data, comorbidities, clinical manifestations, laboratory tests, autoantibodies, and echocardiograms were collected. Body mass index (BMI) was calculated using the formula of weight/height^2 ^(kg/m^2^). Laboratory tests including C-reactive protein (CRP), erythrocyte sedimentation rate (ESR), creatine kinase (CK), CK-MB, lactate dehydrogenase (LDH), cardiac troponin I (cTnI) and N-terminal pro-B-type natriuretic peptide (NT-proBNP) were recorded. MSAs and MAAs were assessed by two commercial semi-quantitative line blot assays (Euroline, Germany).

Transthoracic echocardiography was performed on two-dimensional, pulsed-Doppler and tissue-Doppler modes to evaluate cardiac geometry, blood flow, systolic and diastolic function. Briefly, left atrial diameter (LAD), left ventricular end-systolic diameters (LVESD), left ventricular end-diastolic diameters (LVEDD), left ventricular end-diastolic volume (LVEDV), left ventricular end-systolic volume (LVESV), interventricular septal wall thickness (IVST) and left ventricular posterior wall thickness (LVPWT) were indicative for cardiac volume and wall thickness. Left ventricular ejection fraction (LVEF) was determined as the difference between LVEDV and LVESV, relative to the LVEDV to evaluate systolic function. RWT was determined by the formula: RWT = (IVST + LVPWT)/LVEDD. LV mass was estimated by the formula: LV mass = 0.8 × 1.04 × [(LVEDD + IVST + LVPWT)3 − LVEDD3] + 0.6. LV mass was indexed by body surface area (BSA) (LVMi) calculated at each study time point. BSA was calculated using the formula of 0.0 061 × height (cm) + 0.0128 × weight (kg) − 0.1529. Septal and lateral mitral annular peak early diastolic velocity (e’) recorded by tissue Doppler indicated myocardial diastolic function.

### Assessment of HFpEF-like syndromes

The assessment of HFpEF-like syndromes was based on the HFA-PEFF score proposed by the HFA of the ESC [11]. Briefly, the HFA-PEFF diagnostic algorithm incorporates N-terminal-pro-B-type Natriuretic Peptide (NT-proBNP) levels and echocardiographic parameters including septal and lateral mitral annular peak early diastolic velocity (e’), pulmonary arterial systolic pressure (PASP), tricuspid regurgitation peak velocity, left ventricular global longitudinal systolic strain (GLS), left atrial volume indexed to body surface area (LAVi), left ventricular mass indexed to body surface area (LVMi) and relative wall thickness (RWT). The score contains functional, morphological, and biomarker domains. Within each domain, a major criterion scores 2 points or a minor criterion 1 point. The calculation of the HFA-PEFF score in our cohort was briefly summarized as Venn diagrams (Supplementary Fig. 1).

### Assessment of myositis disease activity

The assessment of disease activity in myositis was according to the core set measures (CSMs): physician global activity (PhGA), patient global activity (PGA), manual muscle testing-8 (MMT-8), health assessment questionnaire (HAQ), myositis disease activity assessment visual analogue scale (MYOACT) [27].

### Statistical analysis

Continuous variables were expressed as the mean and standard deviation (SD) if data were normally distributed or the median with interquartile ranges if not. Normal distribution was evaluated with the Kolmogorov–Smirnov test. Differences in baseline characteristic among three groups were compared by one-way analysis of variance (ANOVA) followed by post hoc Bonferroni test for normally distributed data and by nonparametric test for not normally distributed data. Categorical data were summarized as proportions, and differences were analyzed by Chi-square or Fisher’s exact test. Differences in follow-up echocardiographic parameter changes between patients with positive or negative AMA-M2 and anti-SRP antibody were compared by Student’s unpaired two-tailed t-test.

Spearman’s correlation analysis was conducted to explore the relationship between the HFA-PEFF score and the CSMs. Univariate and multivariate ordinal regression models were performed to screen risk factors for high HFA-PEFF scores. The endpoint of all-cause death was compared in groups stratified by the HFA-PEFF score tertiles, AMA-M2 and anti-SRP antibody with the log-rank test. Survival curves were obtained using the Kaplan–Meier method and censoring variables referred to right censored. Furthermore, cox regression hazard models were also performed to test the independent prognostic value of the HFA-PEFF score after adjusting for confounding risk factors including age, gender, BMI, ILD, serum levels of CK and CK-MB. All statistical analyses were performed with the SPSS 25.0 for Windows (SPSS, Inc., Chicago, IL, USA). A 2-tailed *p* < 0.05 was considered statistically significant. All authors had full access to the data in the study and took responsibility for the integrity of data and accuracy of data analysis.

## Results

### Demographic, clinical, and laboratory characteristics of patients with IIM classified by the HFA-PEFF score

A total of 220 myositis patients without acute cardiovascular events were enrolled for HFpEF risk assessment by virtue of the HFA-PEFF score in this study. The scoring details of each domain were presented as Venn diagrams (Supplementary Fig. 1). By summing up the score of three domains, we found 34 patients (score ≥ 5 points) reached the diagnostic criteria for HFpEF, 107 suspected patients (score 2—4 points) warranted further examinations and only 79 patients (score 0—1 points) were below the pre-alarm value based on the HFA-PEFF score. Thus, a fair proportion of HFpEF was present in patients with IIM.

When dividing the study population into low, intermediate and high tertiles of the HFA-PEFF score, we found patients with higher tertiles tended to be older, comorbid with higher proportions of overweight (*p* = 0.044), hypertension (*p* = 0.001), diabetes mellitus (*p* < 0.001) and present as dyspnea (*p* = 0.029) and palpitation (*p* < 0.001) more in demographic and clinical manifestations. In terms of laboratory indicators, higher-tertile groups had significantly higher levels of LDH (*p* = 0.021), cTnI (*p* = 0.003) and NT-proBNP (*p* < 0.001). No significant differences were found in muscle enzymes such as CK (*p* = 0.979) and CK-MB (*p* = 0.206). Finally, the CSMs of myositis disease activity were compared among groups, and it turned out that patients with higher HFA-PEFF scores had significantly higher PhGA (*p* < 0.001), PGA (*p* < 0.001), HAQ (*p* = 0.058) and MYOACT (*p* = 0.001), but not MMT-8 (*p* = 0.977) (Table 1).

**Table 1:** Baseline characteristics

HFA-PEFF score tertiles	Low (*n* = 79)	Intermediate (*n* = 107)	High *(n* = 34)	*p*-value
Demographics				
Female gender, *n* (%)	59(74.7%)	68(63.6%)	23(67.6%)	0.272
Age, median (IQR), years	50(41–59)	55(48–63)	66(50–68)	0.001
BMI > 24 kg/m^2^, *n* (%)	15(30.0%)	38(42.2%)	39(57.6%)	0.044
Duration, median (IQR), months	5(2–12)	6(3–12)	8(4–15)	0.497
Clinical manifestations, *n* (%)				
Fever	23(29.1%)	54(50.5%)	10(29.4%)	0.006
Rash	49(62.0%)	70(65.4%)	20(58.8%)	0.758
Muscle weakness	39(49.4%)	53(49.5%)	17(50.0%)	0.998
Arthralgia	36(45.6%)	44(41.1%)	7(20.6%)	0.040
Dysphagia	6(7.6%)	9(8.4%)	4(11.8%)	0.764
Dyspnea	30(38.0%)	53(49.5%)	22(64.7%)	0.029
Palpitation	7(8.9%)	32(29.9%)	13(38.2%)	< 0.001
Interstitial lung disease	53(67.1%)	81(75.7%)	30(88.2%)	0.056
Comorbidities, *n* (%)				
Hypertension	8(10.1%)	25(23.4%)	14(41.2%)	0.001
Diabetes mellitus	10(12.7%)	19(17.8%)	15(44.1%)	< 0.001
Malignancies	6(7.6%)	9(8.4%)	4(11.8%)	0.764
Renal dysfunction ^a^	0(0.0%)	1(1.0%)	0(0.0%)	0.567
Laboratory values, median (IQR)				
CK, IU/L	99(41–408)	105(51–397)	80(54–495)	0.979
CK-MB, ng/mL	2.6(0.7–8.4)	2.3(1.1–12.3)	3.5(1.5–14.0)	0.206
LDH, IU/L	245(174–370)	296(220–431)	313(225–497)	0.021
cTnI, ng/mL	0.01(0.01–0.01)	0.01(0.01–0.03)	0.03(0.01–0.05)	0.003
NT-proBNP, pg/mL	68.1(45.2–100.4)	144.3(61.9–298.4)	413.6(212.6–690.2)	< 0.001
Core set measures, median (IQR)				
PhGA	2.0(1.0–2.5)	3.0(2.0–3.5)	4.5(3.5–5.1)	< 0.001
PGA	2.0(1.0–3.5)	3.0(2.0–4.5)	4.5(3.0–6.0)	< 0.001
MMT-8	80(70–80)	80(70–80)	78(74–80)	0.977
HAQ	0.2(0.0–0.4)	0.2(0.0–0.7)	0.3(0.2–1.2)	0.058
MYOACT	3(2–4)	3(2–4)	4(3–4)	0.001

*BMI* Body mass index, *CK* Creatine kinase, *LDH* Lactate dehydrogenase, *cTnI* Cardiac troponin I, *NT-proBNP* N-terminal pro-B-type natriuretic peptide, *PhGA* Physician global activity, *PGA* Patient global activity, *MMT-*8 Manual muscle testing-8, *HAQ* Health assessment questionnaire, *MYOACT* Myositis disease activity assessment visual analogue scale, *IQR* Interquartile range

^a^Renal dysfunction is defined as glomerular filtration rate (eGFR) < 60 ml /min1.73m^2^

**Fig. 2:**
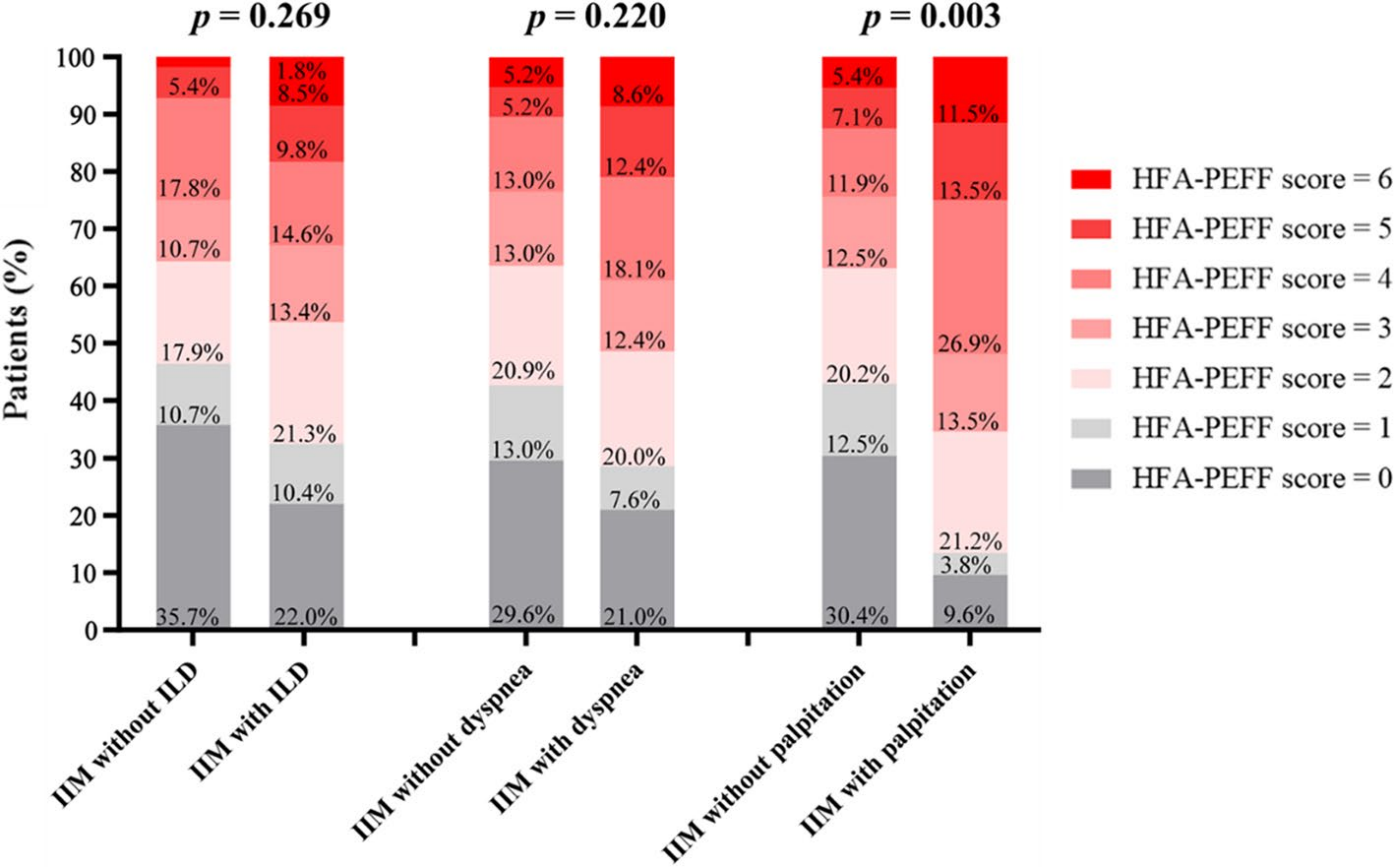
Subgroup analysis of the HFA-PEFF score distribution between myositis patients with or without ILD, dyspnea and palpitation. *IIM* Idiopathic inflammatory myopathy, *ILD* Interstitial lung disease

It was well known that interstitial lung disease (ILD) was a common complication of IIM, and the incidence of ILD was 67.1%, 75.7% and 88.2% respectively in the three groups of our cohort (*p* = 0.056). Unexplained dyspnea may as well be ILD-related in this condition. Hence, in order to rule out the interference of ILD and ILD-related symptoms in HFpEF, subgroup analysis of the HFA-PEFF score distribution between patients with or without ILD and dyspnea was performed. Our results showed that a balanced HFA-PEFF score distribution between myositis patients with or without ILD (*p* = 0.269) and dyspnea (*p* = 0.220). In contrast, significant distribution differences were present between patients with or without palpitation symptoms (*p* = 0.003) (Fig. 2). Therefore, the presence of ILD and dyspnea did not mediate the score distribution.

### The correlation between the HFA-PEFF score and disease activity measurements in IIM patients

Via preliminary comparison of the baseline data, we have known that patients with higher HFA-PEFF scores had higher PhGA, PGA, HAQ, and MYOACT measurements except for the MMT-8 score. Herein, in order to further clarify the association between the HFA-PEFF score and disease activity measurements in patients with IIM, we performed spearman’s correlation analysis. Our result showed that the HFA-PEFF score correlated positively with PhGA (*r* = 0.596, *p* < 0.001), PGA (*r* = 0.405, *p* < 0.001) and MYOACT (*r* = 0.257, *p* < 0.001) measurements. However, no such relationship was found in terms of HAQ (*p* = 0.103) and MMT-8 (*p* = 0.441) (Table 2). Hence, the HFA-PEFF score was parallel to the severity of myositis activity, especially extra-muscle disease activity.

**Table 2:** Spearman’s correlation of HFA-PEFF score with core set measures in patients with idiopathic inflammatory myopathy

Core set measures	Correlation coefficient *r (p—value)*
PhGA	0.596^**^
PGA	0.405**
MMT-8	0.052
HAQ	0.110
MYOACT	0.257**

*PhGA* Physician global activity, *PGA* Patient global activity, *MMT*-8 Manual muscle testing-8, *HAQ* Health assessment questionnaire, *MYOACT* Myositis disease activity assessment visual analogue scale, *HFA-PEFF* Heart Failure Association Pre-test assessment, Echocardiography and natriuretic peptide, Functional testing, Final etiology. **p < 0.01

### The role of MSAs and MAAs in HFpEF in patients with IIM

**Fig. 3:**
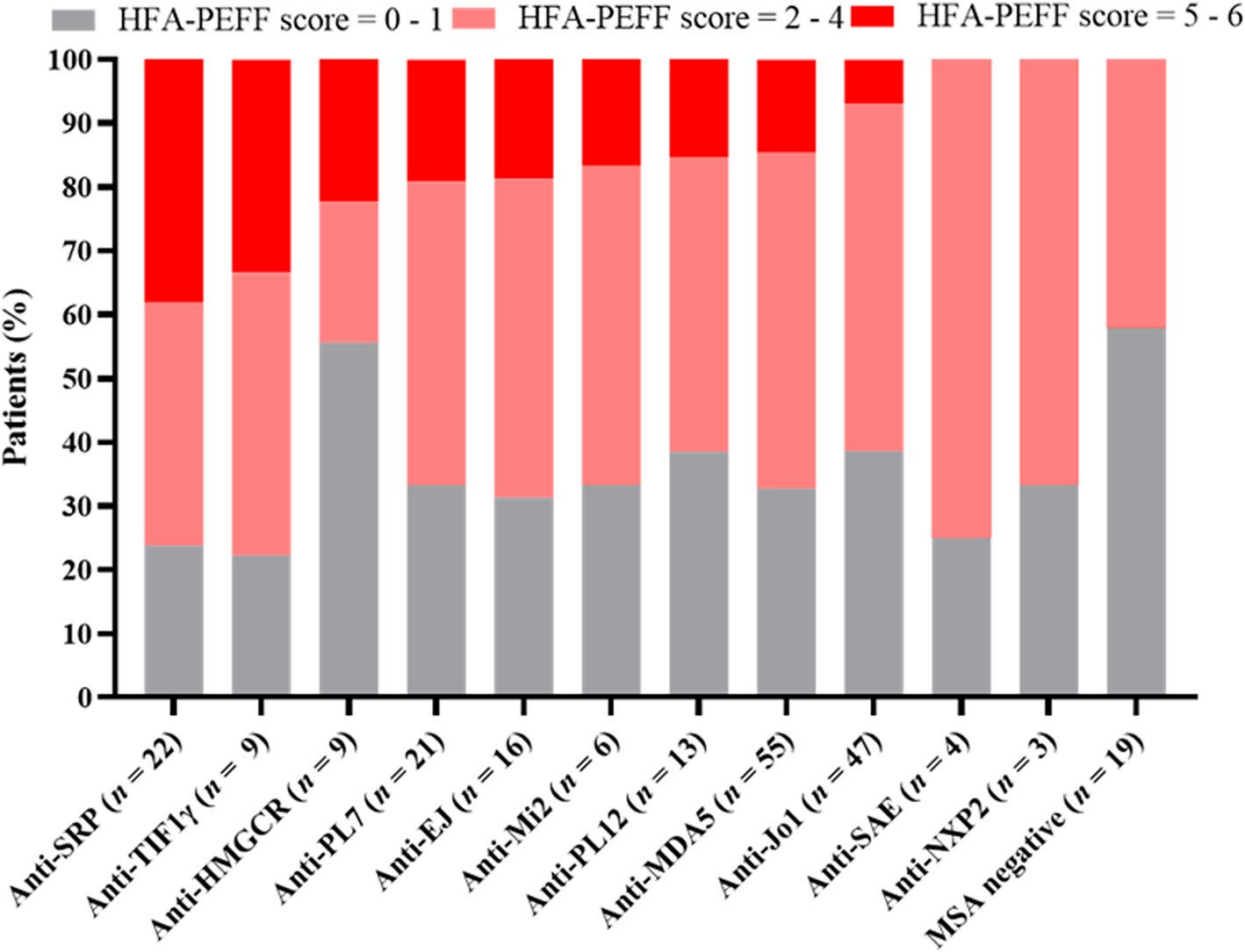
The HFA-PEFF score distribution among subgroups with different positive myositis-specific antibodies. *HFA-PEFF* Heart Failure Association Pre-test assessment, Echocardiography and natriuretic peptide, Functional testing, Final etiology, *MSA* Myositis specific antibodies

In light of the link between MSAs and specific clinical phenotypes, we classified the whole cohort by MSAs to investigate the presence of HFpEF phenotype in each subgroup. Among the 220 patients enrolled in the study, 209 participants had positive MSAs. The distribution discrepancy of the HFA-PEFF score tertiles did exist in MSAs subgroups. Detailed MSAs subgroups were ranked from the highest to the lowest to visualize the high-tertile HFA-PEFF score proportion (Fig. 3). To be mentioned, patients with positive anti-SRP antibody took up the highest proportion (38.1%) of the high-tertile HFA-PEFF score, namely the diagnosed HFpEF. Apart from MSAs, the coexistence of MAAs is also familiar in IIM. Typical MAAs include anti-Ku, anti-PMScl75/100, anti-Ro52 antibodies and AMA-M2. After comparing the positive rate of the forementioned autoantibodies in low, intermediate and high tertiles of the HFA-PEFF score by Chi-square test, AMA-M2 positivity rate turned out to be significantly higher in groups with higher-tertiles HFA-PEFF scores in our cohort (*p* = 0.011) (Table 3). To conclude, patients with AMA-M2 and anti-SRP antibody positivity were more inclined to develop HFpEF to some extent.

**Table 3:** The autoantibody profile of myositis patients and the distribution of HFA-PEFF score tertiles in each subgroup

HFA-PEFF score tertiles	Low	Intermediate	High	*p*-value
Myositis-specific antibodies (MSAs), *n* (%)				
Anti-MDA5	18(32.7%)	29(52.7%)	8(14.5%)	0.335
Anti-Jo1	17(38.6%)	24(54.5%)	3(6.8%)	
Anti-EJ	5(31.3%)	8(50.0%)	3(18.8%)	
Anti-PL7	7(33.3%)	10(47.6%)	4(19.0%)	
Anti-PL12	5(38.5%)	6(46.2%)	2(15.4%)	
Anti-SRP	5(23.8%)	8(38.1%)	8(38.1%)	
Anti-HMGCR	5(55.6%)	2(22.2%)	2(22.2%)	
Anti-TIF1γ	2(22.2%)	4(44.4%)	3(33.3%)	
Anti-Mi2	2(33.3%)	3(50.0%)	1(16.7%)	
Anti-NXP2	1(33.3%)	2(66.7%)	0(0.0%)	
Anti-SAE	1(25.0%)	3(75.0%)	0(0.0%)	
MSA negative	11(57.9%)	8(42.1%)	0(0.0%)	
Myositis-associated antibodies (MAAs), *n* (%)				
Anti-Ku	3(33.3%)	5(55.6%)	1(11.1%)	0.894
Anti-PMScl75/100	8(36.4%)	11(50.0%)	3(13.6%)	0.969
AMA-M2	2(11.1%)	10(55.6%)	6(33.3%)	0.011
Anti-Ro52	37(30.3%)	67(54.9%)	18(14.8%)	0.096

*HFA-PEFF* Heart Failure Association Pre-test assessment, Echocardiography and natriuretic peptide, Functional testing, Final etiology

**Fig. 4:**
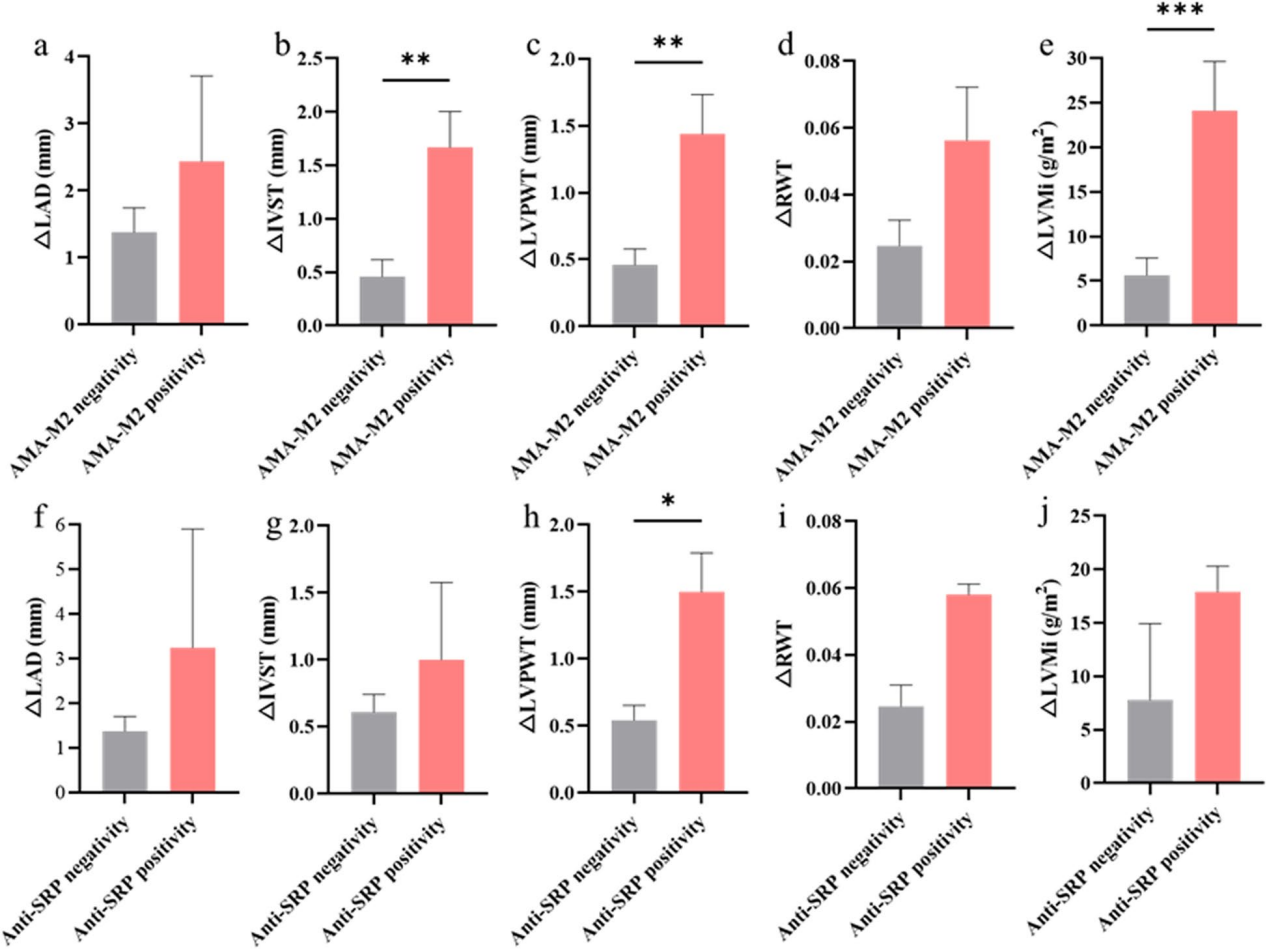
Comparisons of follow-up echocardiographic parameter changes in patients with IIM. The comparisons of (**a**) ΔLAD, (**b**) ΔIVST, (**c**) ΔLVPWT, (**d**) ΔRWT and (**e**) ΔLVMi in patients with positive or negative AMA-M2. The comparisons of (**f**) ΔLAD, (**g**) ΔIVST, (**h**) ΔLVPWT, (**i**) ΔRWT and (**j**) ΔLVMi in patients with positive or negative anti-SRP antibody. All data are presented as mean ± SEM. *p < 0.05, **p < 0.01, ***p < 0.01. *LAD* Left atrial diameter, *IVST* Interventricular septal wall thickness, *LVPWT* Left ventricular posterior wall thickness, *RWT* Relative wall thickness, *LVMi* Left ventricular mass indexed to body surface area

Of the 220 eligible participants, 73 patients had follow-up echocardiographic examinations over a median of 36 months. Considering the HFpEF-predictive role of AMA-M2 and the highest proportion of high-tertile HFA-PEFF scores in anti-SRP myositis patients, we further investigated the morphological and functional changes in echocardiography in these populations (Fig. 4). As a whole, we found patients with positive AMA-M2 or anti-SRP antibody all manifested a more obvious inclination towards concentric hypertrophy characterized by increases in LAD, IVST, LVPWT, RWT and LVMi. To be specific, groups with positive AMA-M2 developed significantly more increase in IVST (*p* < 0.01) (Fig. 4b), LVPWT (*p* < 0.01) (Fig. 4c), LVMi *(p* < 0.001) (Fig. 4e) compared to groups with negative AMA-M2. Additionally, differences were present in △LVPWT (*p* < 0.05) (Fig. 4h) between groups with positive or negative anti-SRP antibodies.

### Risk factors assessment for HFpEF in myositis patients

Via above preliminary analysis, we found HFpEF manifestations were prevalent in IIM and might predispose to specific population. Herein, we further investigated risk factors for HFpEF in myositis patients by performing the ordinal regression for the graded HFA-PEFF score (Table 4). In univariate regression, elderly, higher BMI, hypertension, diabetes mellitus, interstitial lung disease, dyspnea symptom, palpitation symptom, anti-SRP antibody positivity, AMA-M2 positivity, elevated serum levels of LDH and cTnI (all *p* < 0.05) were all responsible for higher-grade HFA-PEFF score. In multivariate regression, palpitation symptoms (*OR*: 2.59(1.35—4.99), *p* = 0.004), AMA-M2 positivity (*OR*: 3.80(1.49—9.68), *p* = 0.005), elevated LDH level (*OR*: 3.59(1.86—6.92), *p* < 0.001) and elevated cTnI level (*OR*: 4.43(2.03—9.65), *p* < 0.001) were independently predictive for the HFpEF phenotype after adjusting for confounding clinical risk factors including age, BMI, hypertension, diabetes and interstitial lung disease.

**Table 4:** Ordinal regression models for high HFA-PEFF scores in myositis patients

	Univariate		Multivariate	
Covariates	OR (95% CI)	*p*—value	OR (95% CI)	*p*—value
Age	1.03 (1.01–1.05)	0.001		
Body mass index	1.07(1.00–1.15)	0.045		
Hypertension	2.91(1.63–5.20)	< 0.001		
Diabetes mellitus	2.44(1.35–4.41)	0.003		
Interstitial lung disease	1.81(1.05–3.11)	0.033		
Dyspnea symptom	1.87(1.64–2.99)	0.010	1.52(0.83–2.76)	0.175
Palpitation symptom	3.24(1.86–5.64)	< 0.001	2.59(1.35–4.99)	0.004
Anti-SRP antibody	2.53(1.04–6.16)	0.041	1.89(0.82–4.36)	0.133
Anti-TIF1γ antibody	1.69(0.53–5.36)	0.374		
Anti-HMGCR antibody	0.55(0.15–2.04)	0.371		
Anti-synthetase antibody	0.94(0.59–1.51)	0.806		
Anti-Mi2 antibody	0.73(0.18–3.01)	0.659		
Anti-MDA5 antibody	1.18(0.69–1.99)	0.549		
AMA-M2	4.13(1.72–9.92)	0.001	3.80(1.49–9.68)	0.005
Elevated CK level	0.98(0.58–1.64)	0.923		
Elevated LDH level	2.39(1.33–4.27)	0.003	3.59(1.86–6.92)	< 0.001
Elevated cTnI level	4.31(2.14–8.67)	< 0.001	4.43(2.03–9.65)	< 0.001

*CK* Creatine kinase, *LDH* Lactate dehydrogenase, *cTnI* Cardiac troponin I, *OR* Odd ratio, *CI* Confidence interval

### The prognostic value of the HFA-PEFF score in patients with IIM

**Fig. 5:**
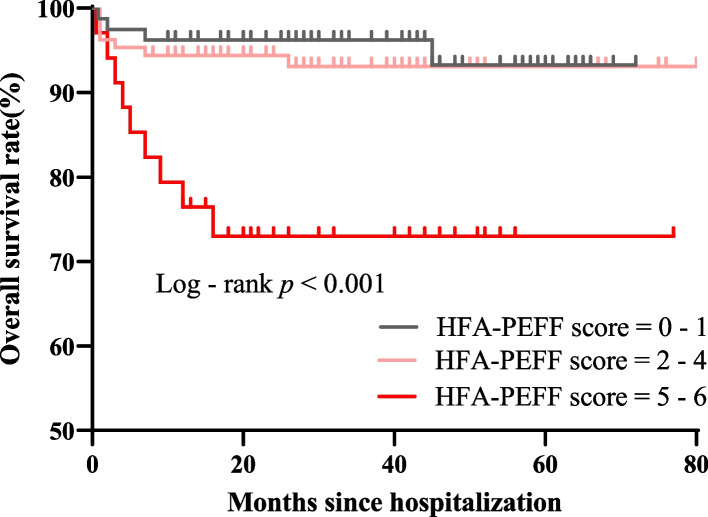
Kaplan–Meier survival curves among myositis patients with different HFA-PEFF score tertiles. *HFA-PEFF* Heart Failure Association Pre-test assessment, Echocardiography and natriuretic peptide, Functional testing, Final etiology

**Fig. 6:**
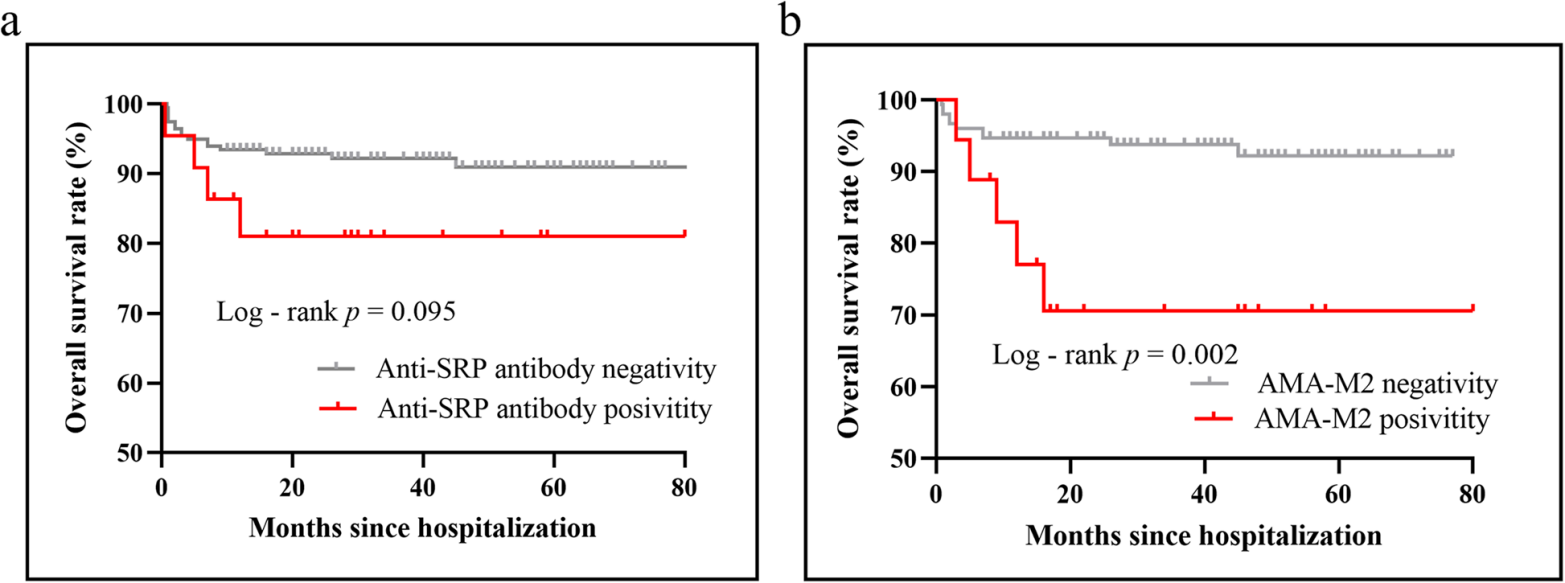
Kaplan–Meier survival curves in myositis patients with AMA-M2 or anti-SRP antibody. Kaplan–Meier survival curves between myositis patients with (**a**) positive or negative AMA-M2 and (**b**) positive or negative anti-SRP antibody

Last but not least, we explored whether the HFpEF-like syndrome assessed by the HFA-PEFF score was related to the overall prognosis in IIM. A total of 20 patients reached the composite endpoint of all-cause death during a median follow-up of 36 months. Kaplan–Meier survival curves exhibited significant differences among the low, intermediate and high-tertiles of HFA-PEFF score (*p* < 0.001) (Fig. 5). Every point accumulation in the HFA-PEFF score from 0 to 6 points corresponded to a 100% increase in the mortality risk after adjusting for confounding risk factors including age, gender, BMI, ILD, serum levels of CK and CK-MB (*HR*: 2.00 (1.31—3.08), *p* = 0.001). Moreover, compared to patients with negative AMA-M2, patients with positive AMA-M2 had significantly lower overall survival rates (*p* = 0.002) (Fig. 6a), which might as well be ascribed to high HFpEF proportions in this population as mentioned above. To be mentioned, patients with positive anti-SPR antibody also presented with a tendency towards relatively poorer outcomes though the survival discrepancy between groups remained insignificant (*p* = 0.095) (Fig. 6b).

## Discussion

Multiple autoimmune diseases, IIM included, were reported to be casual for HFpEF since immunity and inflammation were overlapping risk factors for both disorders. Nonetheless, the diagnosis of early-stage HFpEF without decompensated manifestations remained challenging. In the present study, we epidemiologically elaborated on the prevalence of HFpEF in IIM via the HFA-PEFF score. To the best of our knowledge, it was the first study that adopted the well-recognized score to reveal the prevalence of HFpEF in rheumatologic diseases.

As a subtype of heart failure with heterogeneous manifestations, HFpEF has gradually raised concern in recent years. Common risk factors for HFpEF include obesity, hypertension, diabetes mellitus, elderly and atrial fibrillation [28]. In our cohort, the comorbidity rate of overweight, hypertension and diabetes mellitus in the high-tertile HFA-PEFF group was higher, which was consistent with the previous studies. Besides, palpitation symptom, AMA-M2 positivity and elevated serum levels of LDH, cTnI were independent risk factors for HFpEF in the multivariate regression analyses. Moreover, patients with unexplained dyspnea are highly suggestive for HFpEF [13]. Since a fair proportion of myositis patients manifest as ILD and ILD-related dyspnea, it was sensible to be beware of the presence of HFpEF-associated breathlessness covered up by ILD-related breathlessness in IIM. Subgroup analysis in our study revealed a balanced distribution of the HFA-PEFF score between myositis patients with or without ILD and dyspnea. So, it was indispensable to recognize occult or misdiagnosed cardiac dyspnea related to HFpEF in the case of IIM.

Previous research demonstrated that high HFA-PEFF scores could rule in HFpEF with 93% specificity and low scores could rule out HFpEF with 99% sensitivity [18]. Although we could not make a definite diagnosis of HFpEF merely based on the scoring points, it was of significance to briefly estimate the disease burden of HFpEF in IIM via a non-invasive and convenient tool. In our study, it turned out quite a number of myositis patients were at middle-to-high risk for HFpEF, which deserved matched attention. Moreover, early recognition of potential HFpEF by the screening HFA-PEFF score was helpful for preventing progression towards overt heart failure through early intervention and management.

In our study, we found the HFA-PEFF score positively correlated to myositis disease activity measurements including PGA, PhGA and MYOACT other than MMT-8. Our results suggested that the HFA-PEFF score possessed a certain value to reflect global disease activity. It could be speculated that myositis patients with HFpEF would manifest as severer myositis manifestations and accordingly had a relatively higher mortality rate. From another perspective, no relationship existed between HFpEF and muscle weakness in our cohort. The relationship between skeletal and cardiac muscle involvement in IIM was hotly debated [29, 30]. In line with our study, several CMR-based prospective studies revealed significant skeletal and cardiac muscle pathologic changes including edema and fibrosis in IIM patients, while no linear relationship existed between pathological changes in cardiac and skeletal muscles [31, 32]. Since acute inflammation and diffuse fibrosis of the myocardium are important characteristics of HFpEF, we supposed that systematic inflammation conditions could breed myocardial pathologic changes in IIM. Hence, HFpEF was indeed an outcome of systemic inflammation rather than local muscle lesions.

To be mentioned, we preliminarily investigated the HFpEF-predictive role of MSAs and MAAs. Anti-SRP antibody is recognized as a marker of immune-mediated necrotizing myositis. Patients with positive anti-SRP antibody were reported to be susceptible to cardiac involvement in the form of myositis, arrhythmia and cardiomyopathy in early years while subsequent studies came to controversial conclusions [6, 33, 34]. AMA-M2 typically represents the hallmark of primary biliary cirrhosis but has been increasingly observed in IIM [35, 36]. Maeda et al. estimated the prevalence of AMA-M2 in 212 myositis patients at around 11.3% and the proportion of myocardial involvement in patients with positive AMA-M2 at over 33.3% [36]. Lixi et al. revealed that patients with positive AMA-M2 were five times more likely to be comorbid with cardiac complications after adjusting for confounding risk factors [37]. Nonetheless, the definition of cardiac involvement in prior studies was broad and ambiguous and the HFpEF-predictive role of autoantibodies was scarcely explored. In our study, we identified AMA-M2 as a potential risk factor for HFpEF and poor prognosis. Additionally, patients with positive anti-SRP antibody exhibited the highest probability of developing HFpEF and a relatively lower overall survival rate as well. Nonetheless, whether the HFpEF-predictive role of AMA-M2 and anti-SRP antibody was merely an epiphenomenon or existing underlying pathogenic mechanisms required further exploration. It was assumed that AMA-M2 might contribute to HFpEF by targeting the inner heart mitochondrial membrane protein, thus impairing the phosphorylation and oxidative capacity of mitochondria [38]. While the pathogenicity of anti-SRP antibody against myocardium remained unknown. Cécile et al. demonstrated that anti-SRP antibody played a pathogenic role through a complement-mediated mechanism based on an in vivo study when it came to its pathogenicity towards skeletal muscle necrosis [39]. Whether cardiac and skeletal muscles shared similar pathological changes need further investigation. Analogously, anti-Ro52 antibodies were proved to exert their pathogenic effect by cross-reacting with a molecule in the fetal heart to cause congenital heart block in a rat model [40]. However, no anti-Ro52-related cardiac involvement was found in our study yet.

Finally, we explored the prognostic value of the HFA-PEFF score in the myositis cohort for the first time. Higher HFA-PEFF scores have been proven to be predictive of increased risk for heart failure hospitalization or death in the large DIAST-CHF and ARIC studies [23, 24]. Besides, Daniela et al. verified the prognostic value of the HFA-PEFF score in cardiac amyloidosis, a specific etiology of HFpEF recently [22]. Moreover, Yannis et al. pointed out that the HFA-PEFF score could serve as an independent prognostic predictor in cirrhosis patients susceptible to cirrhotic cardiomyopathy [41]. In consistent with these recent studies, myositis patients with higher-tertile HFA-PEFF scores did suffer from poorer prognosis in our study. Furthermore, the HFA-PEFF score was an independent risk factor for all-cause death in patients with IIM. In short, myositis patients comorbid with HFpEF suffered from a significantly poor prognosis.

We appreciate the limitations in our study. First, this was a single-center retrospective study based on prospectively collected data. Second, follow-up data are not intact with limitations to all-cause death and partially recorded echocardiographic examinations. Long-term regular follow-up of myositis patients is warranted.

## Conclusions

HFpEF is prevailing in patients with IIM. The HFA-PEFF score held great diagnostic and prognostic value for HFpEF in IIM. In view of the profound impact of HFpEF on myositis disease activity and overall survival, it was of critical significance to reinforce the awareness of screening and management of HFpEF in IIM, especially in patients with AMA-M2 and anti-SRP antibody.

### Supplementary Information

**Additional file 1: Supplementary fig 1.**

## Data Availability

All data and materials generated or analysed during this study are included in this published article (and its supplementary information files).

## Abbreviations

HFpEF: Heart failure with preserved ejection fraction
IIM: Idiopathic inflammatory myopathy
HFA-PEFF: Heart Failure Association Pre-test assessment, Echocardiography and natriuretic peptide, Functional testing, Final etiology.
MSA: Myositis-specific antibody
MAA: Myositis-associated antibody
DM: Dermatomyositis
PM: Polymyositis
IMNM: Immune-mediated necrotizing myopathy
IBM: Inclusion body myositis
HFrEF: Heart failure with reduced ejection fraction
CSMs: Core set measures
PhGA: Physician global activity
PGA: Patient global activity
MMT-8: Manual muscle testing-8
HAQ: Health assessment questionnaire
MYOACT: Myositis disease activity assessment visual analogue scale
ILD: Interstitial lung disease
