# Supplementary material for: Value of the HFA-PEFF diagnostic algorithms for heart failure with preserved ejection fraction to the inflammatory myopathy population
Source: Arthritis Res Ther. 2023 Aug 4;25:141. doi: 10.1186/s13075-023-03131-6 (PMC10401815; doi:10.1186/s13075-023-03131-6)
Supplement: Supplementary file 1 — Additional file 1: Supplementary fig 1. [file 13075_2023_3131_MOESM1_ESM.docx]

**Supplementary information files**


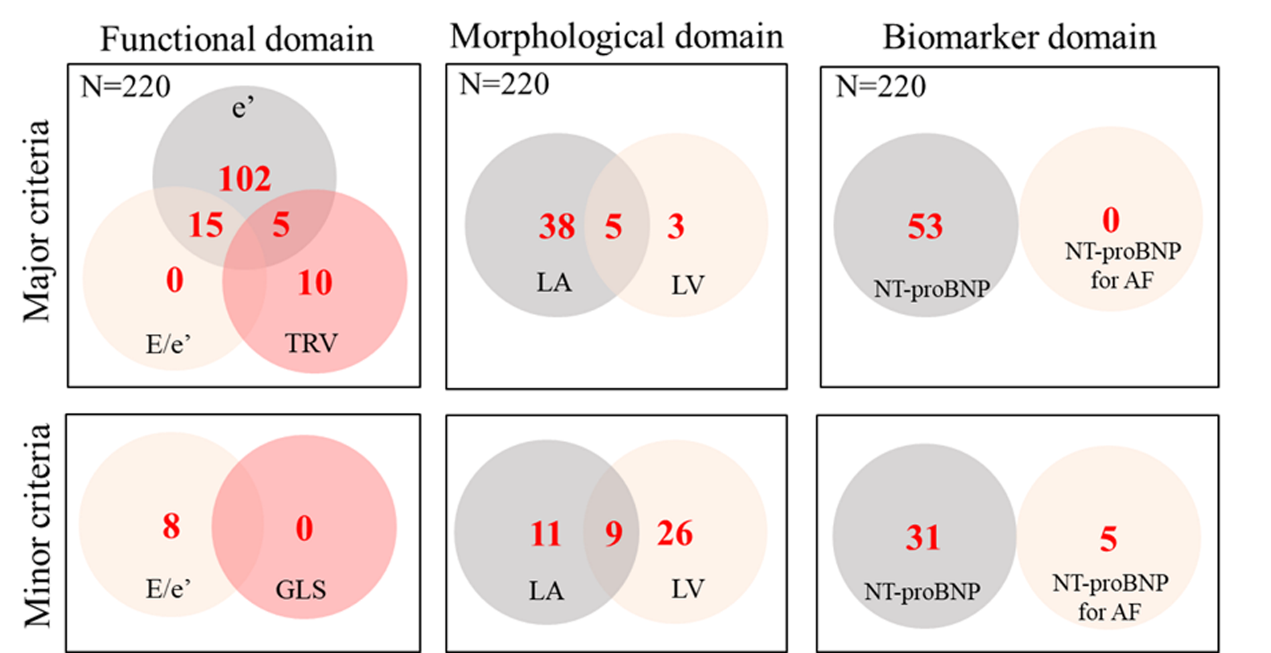


**Figure S1.** Venn diagrams showing the number of patients who met each major and minor criterion in the functional, morphological and biomarker domains of the HFA-PEFF scoring system. TRV, tricuspid regurgitation velocity; LA, left atrium; LV, left ventricle; NT-proBNP, N-terminal pro-B-type natriuretic peptide; AF, atrial fibrillation.
